# Nonatherosclerotic Giant Right Coronary Artery Aneurysm

**DOI:** 10.21470/1678-9741-2020-0649

**Published:** 2022

**Authors:** Lucas Figueredo Cardoso, Ricardo Ribeiro Dias, Lea Maria Macruz Ferreira Demarchi, Lucas Molinari Veloso da Silveira, Charles Mady, Fabio B Jatene

**Affiliations:** 1 Department of Cardiovascular Surgery, Instituto do Coração (InCor), Faculdade de Medicina, Universidade de São Paulo, São Paulo, São Paulo, Brazil.; 2 Laboratory of Anatomic Pathology, Instituto do Coração (InCor), Faculdade de Medicina, Universidade de São Paulo, São Paulo, São Paulo, Brazil.; 3 Department of Cardiology, Instituto do Coração (InCor), Faculdade de Medicina, Universidade de São Paulo, São Paulo, São Paulo, Brazil.

**Keywords:** Coronary Vessels, Coronary Aneurysm, Coronary Artery Bypass, Tomography, X-Ray Computed, Incidental Findings

## Abstract

We present an unusual case of a 67-year-old woman with an incidental finding of a cardiac mass on a chest computed tomography. Coronary angiotomography confirmed the diagnosis of right coronary artery aneurysm, with 5.7×5.7 cm. The patient underwent aneurysm resection and coronary bypass surgery, with subsequent histologic study suggestive of arteritis sequelae. Giant coronary artery aneurysms have a high risk of complications and aneurysm exclusion must be beneficial. This is a rare condition that can also be part of a systemic inflammatory disease.

**Table:** 

Abbreviations, acronyms & symbols	
a	= Coronary adventitia
CT	= Computed tomography
I + M	= Intimal and medial layers
L	= Coronary lumen
RCA	= Right coronary artery

## CASE PRESENTATION

A 67-year-old woman was referred to our hospital due to an incidental finding of a cardiac mass on a chest computed tomography (CT) during investigation of sudden dyspnea. She had normal sinus rhythm, with no ST-segment changes on electrocardiography.

## DISCUSSION

**Fig. 1:**
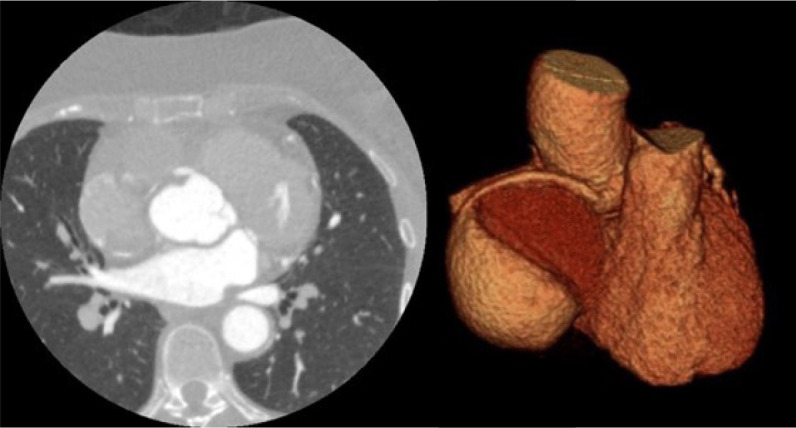
A) Coronary computed tomography angiography showing a saccular aneurysm with a partially calcified mural thrombus. B) Three-dimensional reconstruction confirming that the mass was supplied by the right coronary artery.

Transthoracic echocardiography revealed a 6.3×5.9 cm anechoic mass, partially compressing the right chambers; cardiac function was normal, and no ventricular akinesia was found. Coronary angiotomography showed a saccular aneurysmal dilatation in the middle segment of the right coronary artery (RCA), with 5.7×5.7 cm, associated with a calcified mural thrombus (Figure 1), without significant coronary stenosis. Investigation with whole-body CT scan and cerebral magnetic resonance imaging excluded other vessel aneurysms.

**Fig. 2:**
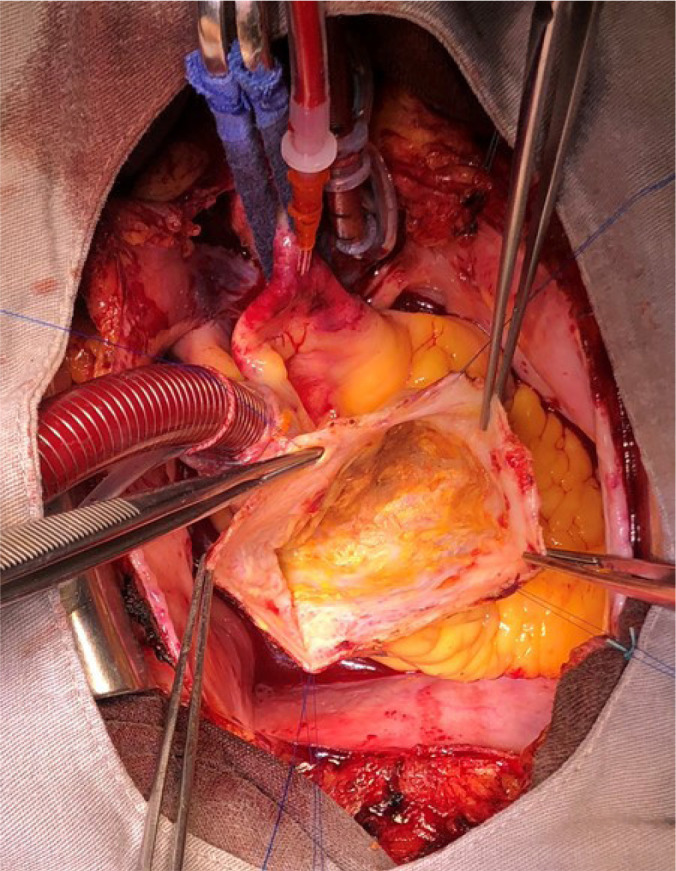
Intraoperative view of aneurysm exclusion.

**Fig. 3:**
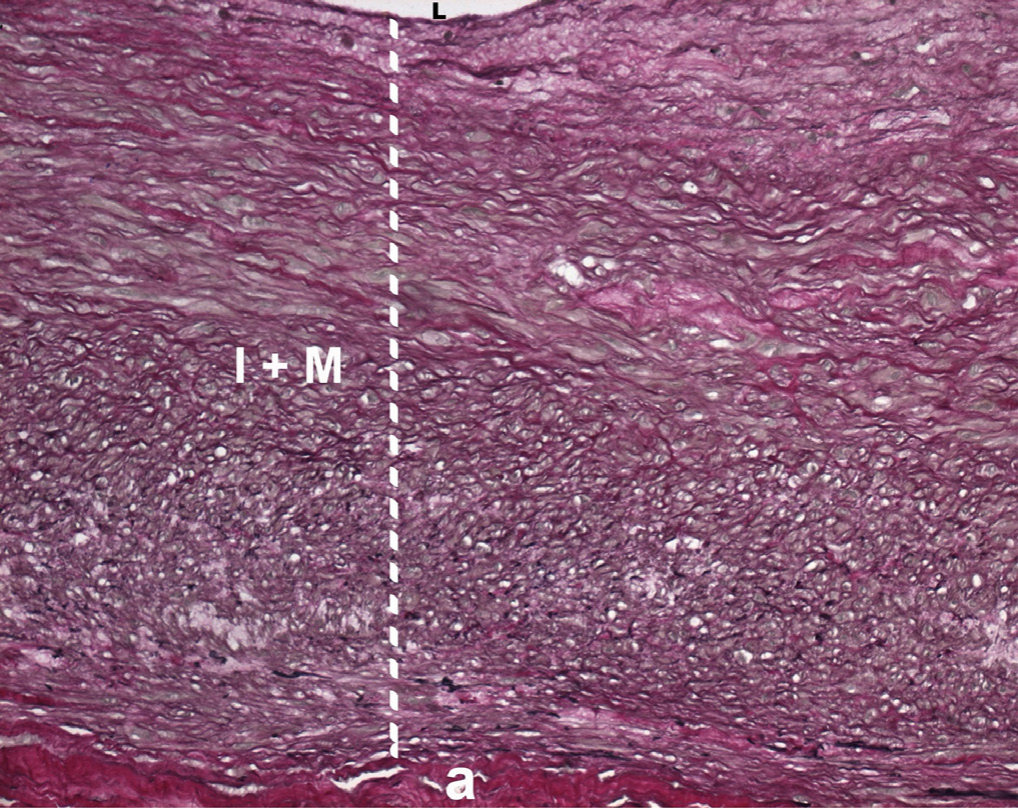
Photomicrograph of right coronary artery with sequelae of arteritis. Destruction of the elastic laminae (black color). a=coronary adventitia; I + M=intimal and medial layers; L=coronary lumen

The patient underwent surgery through full sternotomy, with resection of a giant RCA aneurysm and exclusion of both entrance and exit ostia (Figure 2). Coronary artery bypass surgery was performed with a saphenous vein graft to the RCA. Histologic study was suggestive of arteritis sequelae represented by diffuse thickening of intimal and medial layers with fibrosis, and destruction of the elastic laminae (Figure 3).

## CONCLUSION

Patients with giant coronary artery aneurysms are at high risk of complications and must benefit from aneurysm exclusion^[1]^. This is a rare condition, and atherosclerosis accounts for half of the cases in adults; however, it may also be part of a systemic inflammatory disease^[2]^, as presented in this case.

**Table:** 

Authors' roles & responsibilities	
LFC	Substantial contributions to the acquisition of data for the work; drafting the work; final approval of the version to be published
RRD	Substantial contributions to the analysis of data for the work; revising the work critically for important intellectual content; final approval of the version to be published
LMMFD	Substantial contributions to the acquisition and analysis of data for the work; final approval of the version to be published
LMVS	Substantial contributions to the acquisition of data for the work; drafting the work; final approval of the version to be published
CM	Substantial contributions to the analysis of data for the work; revising the work critically for important intellectual content; final approval of the version to be published
FBJ	Substantial contributions to the analysis of data for the work; revising the work critically for important intellectual content; final approval of the version to be published
